# Long-term response to immunotherapy in patients with hypertrophic pachymeningitis

**DOI:** 10.20407/fmj.2021-026

**Published:** 2022-05-25

**Authors:** Mayumi Senda, Akihiro Ueda, Mizuki Ito, Sayuri Shima, Yasuaki Mizutani, Tatsuro Mutoh, Hirohisa Watanabe

**Affiliations:** 1 Department of Neurology, Fujita Health University, School of Medicine, Toyoake, Aichi, Japan; 2 Department of Neurology, Fujita Health University Bantane Hospital, Nagoya, Aichi, Japan

**Keywords:** Hypertrophic pachymeningitis, Prognosis, Prednisolone, Immunosuppressant, Recurrence

## Abstract

**Objective:**

In this study, we aimed to clarify the relationship between initial treatment response, prednisolone (PSL) dosage, clinical type, and recurrence in patients with hypertrophic pachymeningitis (HP).

**Methods:**

The study cohort comprised eight patients with HP who had been admitted to our hospital from April 2015 to June 2020. Diagnostic criteria for HP included neurological abnormalities and dural thickening on magnetic resonance gadolinium-enhanced T1-weighted images.

**Results:**

Relevant characteristics of the eight study patients are as follows. There were two men and six women. The average age at onset was 58.3 (range: 29–79) years. Three of them had myeloperoxidase-antineutrophil cytoplasmic antibody-related vasculitis, one immunoglobulin G4-related disease, and one ulcerative colitis. The remaining three patients had idiopathic HP. The average maximum dosage of PSL was 0.79 mg/kg/day, and the average daily maintenance dosage 0.18 mg/kg/day. Three patients needed additional immunosuppressive drugs. Both idiopathic and secondary HP initially responded well to PSL, with improvement in activities of daily living. Six patients had some sequelae related to cranial nerve involvement. No relapses occurred while the patients were taking moderate doses of PSL; however, all patients with idiopathic HP had recurrences when their PSL dosage was reduced.

**Conclusions:**

Patients with idiopathic HP and HP associated with immune disorders respond to steroids and immunosuppressive drugs and recover well. However, there is a high rate of relapse after reduction of PSL dosage, mainly in those with idiopathic HP.

## Introduction

Hypertrophic pachymeningitis (HP), a chronic inflammatory disorder, is characterized by partial or diffuse thickening of the dura mater of the brain and spinal cord.^1–4^ An epidemiological study of HP in Japan^1^ found a crude prevalence of 0.949 per 100,000, indicating that this condition is very rare. In HP, increased intracranial pressure and dura mater inflammation can cause headache, direct pressure, and inflammatory spillover from dural thickening. Of note, headache is the first symptom in approximately one-third of cases, occurring in more than 70% of affected individuals at some stage during the course of the disease.^1^ Dural biopsy used to be the gold standard for diagnosis. However, in recent years, a combination of magnetic resonance imaging (MRI), serological and spinal fluid tests, and other investigations to identify background diseases enables a correct diagnosis without an invasive procedure.

HP is classified as idiopathic (no identified cause) or secondary to inflammatory diseases such as immunoglobulin G4 (IgG4)/multifocal fibrosclerosis-related diseases,^5–7^ antineutrophil cytoplasmic antibody (ANCA)-related vasculitis,^8,9^ granulomatosis with polyangiitis,^10^ sarcoidosis,^11^ and rheumatoid arthritis^12,13^ or infectious diseases such as bacterial and fungal infections, including tuberculosis.^14,15^ Neoplastic diseases, including lymphoma^16^ and histiocytosis, and cerebrospinal fluid (CSF) hypovolemia,^17^ can also cause dural thickening. Accurate differentiation is therefore required. In a previous study, the proportions of patients with idiopathic HP, HP with ANCA, and IgG4/multifocal fibrosclerosis-related HP were 44%, 30.2%, and 3.8%, respectively.^1^

After carefully excluding infections, neoplasms, and CSF hypovolemia, methylprednisolone pulse therapy followed by oral prednisolone (PSL) is the initial treatment of choice for HP.^18^ Initial treatment with PSL achieves responses in 87.2% of patients with HP, 92.6% of whom show further improvement with additional immunosuppressive drugs.^1^ Of note, we have previously reported, on the basis of our three cases and a review of 66 reported cases, that recurrence may occur after reduction of the PSL dosage to under 20 mg/day.^19^ However, despite long-term follow-up, relationships between background diseases, PSL dosage, and clinical characteristics and recurrence have not yet been established.

In this study, we studied the clinical courses of 8 of 14 patients with immune-mediated or idiopathic HP who had undergone long-term follow-up, our aim being to clarify the relationships between initial treatment response, PSL dosage, clinical type of HP, and recurrence.

## Methods

### Patient enrollment

**Figure 1:**
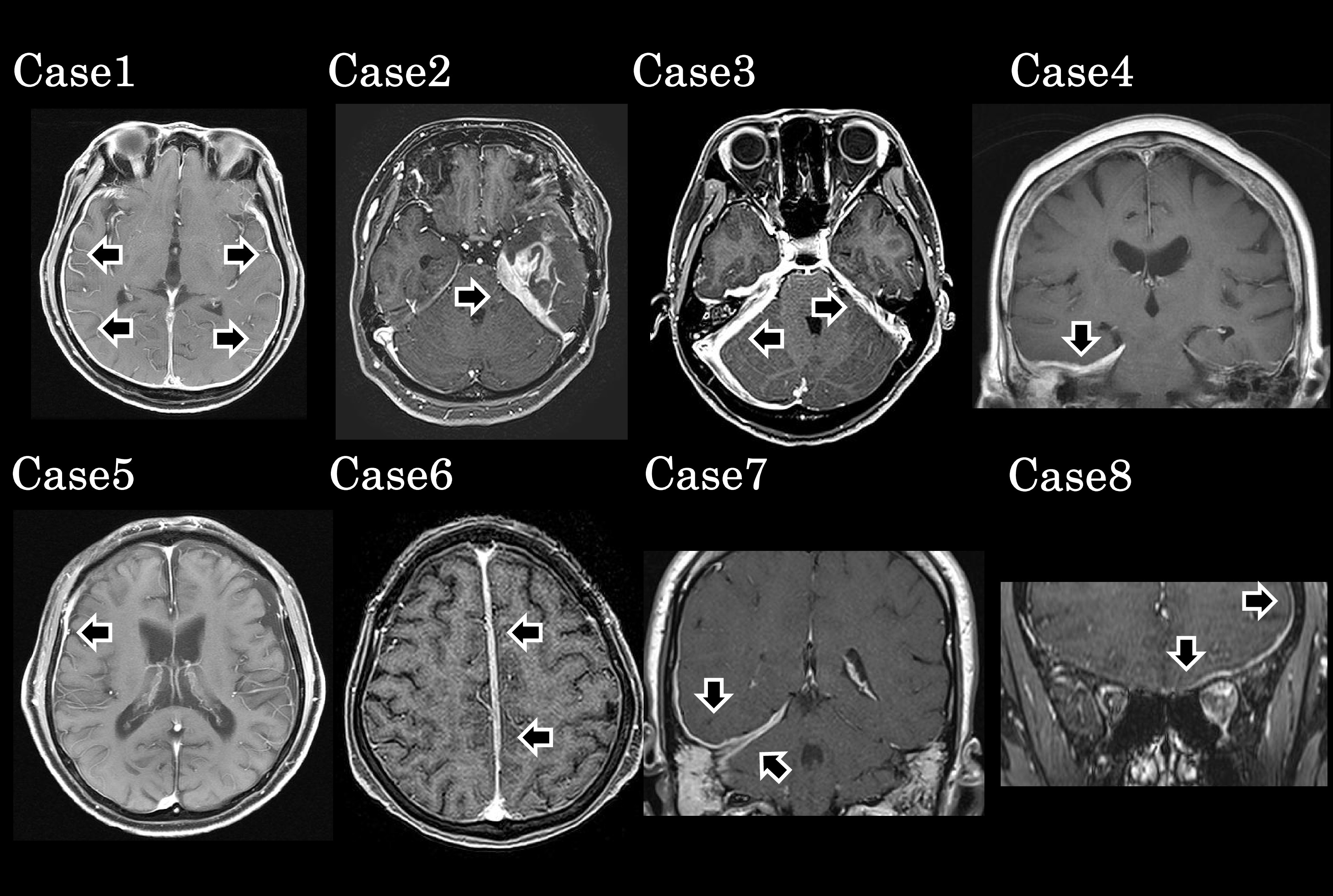
Magnetic resonance imaging findings on gadolinium-enhanced T1-weighted images of our eight patients with acute phase hypertrophic pachymeningitis.

We retrospectively reviewed the medical records of 14 Japanese patients with HP who had undergone detailed evaluation in our department from April 2015 to June 2020. The protocol for this study was approved by the ethics committee of Fujita Health University Hospital (HM21-292). As previously reported,^1^ we defined HP as thickening of the cranial or spinal dura mater on MRI T1 sequences with contrast enhancement (Figure 1) that cannot be attributed to intracranial hypotension, neoplastic pachymeningitis, or infectious diseases. We also excluded patients with dura mater thickening confined to the orbital apex (orbital apex syndrome) or cavernous sinus (cavernous sinus syndrome).

We excluded 6 of the 14 patients because of infection (n=3; two with aspergillus and one with atypical mycobacterium), neoplastic disease (n=1), CSF hypovolemia (n=1), and undetermined cause with rapid progression and death (n=1). All patients with infections were successfully treated with antimicrobial treatments. The two patients with neoplasms and one with CSF hypovolemia were only followed-ups for a short time.

### Clinical features

The mean duration of follow-up of the eight patients was 63.8±56.8 (range: 14–152) months. The variables investigated comprised average age at onset, sex, underlying disease, initial symptoms, and presence or absence of headache during the entire course.

### Laboratory findings

White blood cell (WBC) count, C-reactive protein (CRP), CSF cell count, protein, and glucose were assessed at the first visit. Serological studies included antinuclear antibodies, myeloperoxidase (MPO)-ANCA, proteinase-3-ANCA, and angiotensin-converting enzymes. Enzyme-linked immunosorbent assays were performed to measure ANCA, HP with ANCA being diagnosed if either MPO-ANCA, proteinase-3-ANCA, or ANCA-related angiitis was detected. We diagnosed IgG4-related HP on the basis of established criteria^20^ for IgG4-related disease or evidence of multifocal fibrosclerosis, such as retroperitoneal fibrosis, mediastinal fibrosis, sclerosing pancreatitis, Riedel’s thyroiditis, and pseudotumor of the orbit. Idiopathic HP comprised cases with no evidence of ANCA or IgG4-related conditions or other causes, including relevant autoimmune diseases.

### Radiological findings

MRI was performed using a 1.5 T or 3.0 T scanner (Canon Medical Systems; Otawara city, Japan), 5-mm-thick slices being obtained. A combination of thickened areas with T1 hypointensity and localized, multiple, or diffuse enhancing dural thickening are considered characteristic of HP.^21^ Thus, we evaluated the presence or absence of dura mater thickening on conventional T1-weighted imaging, then categorized MRI enhancement by location, distribution, and pattern in accordance with previously published criteria.^22^ The locations were identified by two experienced neurologists (MI and AU). Diffuse distribution was defined as the contrast area exceeding 50% of the total brain area and focal distribution as contrast area less than 50% of the total area.

### Treatment and prognosis

We evaluated the maximum steroid daily dose converted into PSL, daily maintenance dose of PSL, presence or absence of steroid pulse therapy and immunosuppressive drug combinations, rate of terminating drug treatment, changes in modified Rankin Scale (mRS) score (0, no symptoms; 1, no significant disability despite symptoms; 2, slight disability; 3, moderate disability; 4, moderately severe disability; and 5, severe disability) before and after treatment, presence or absence of sequelae, recurrence rate and symptoms on recurrence, and mortality rate. We defined response to treatment as improvement in radiological findings, amelioration of clinical manifestations, including headache and cranial neuropathies, or resolution of vasculitis in any organ system, in accordance with the European League Against Rheumatism recommendations.^23^

## Results

### Clinical characteristics

The clinical and other relevant characteristics of the eight patients with immune-mediated or idiopathic HP are summarized in Table 1. The study cohort comprised two men and six women of average age 58.3±20.1 (range: 29–79) years. The underlying diseases were as follows: three patients (37.5%) had idiopathic HP, three (37.5%) HP with ANCA, one (12.5%) IgG4-related disease, and one (12.5%) ulcerative colitis. The mean duration of follow-up was 59.3±56.9 months. Headache was the most common symptom at the first visit, being present in seven patients (87.5%). All patients reported headaches at some stage during their clinical course (100%). Other initial symptoms included diplopia in six patients (75%); deafness in four (50%); dizziness, ptosis, and facial pain in two (25%); and visual disturbance, facial sensory disturbance, facial palsy, dysarthria, and dysphasia related to cranial nerve involvements in one (12.5%).

### Radiological and laboratory findings

All patients showed dura mater thickening on conventional T1-weighted imaging and gadolinium-enhanced MRI, the thickening being diffuse in four cases (50%) and focal in the other four (50%). All three patients with HP with ANCA had the focal type of dural thickening, whereas two of the three patients with idiopathic HP and one with IgG4-related HP had diffuse dural thickening.

Six cases (75%) had high WBC counts and all (100%) had high CRP concentrations at the first visit. Antinuclear antibody was positive in three cases (37.5%). No patient with HP with ANCA had antinuclear antibodies, whereas two of the three with idiopathic HP tested positive for these antibodies. As for CSF findings, seven patients (87.5%) showed pleocytosis and seven (87.5%) high protein concentrations, whereas none of them had low CSF glucose concentrations.

### Treatment and long-term follow-up

All patients were treated with PSL. Steroid pulse therapy was administered during the acute phase in six of the eight cases (75%): two of the three with idiopathic HP, two of the three with HP with ANCA, the one with IgG4-related HP, and the one with ulcerative colitis-related HP case. The average maximum PSL dosage was 0.79 mg/kg/day, and the daily maintenance PSL dosage 0.18 mg/kg/day. Three patients needed additional immunosuppressive drugs. All immunosuppressive treatments achieved improvements in mRS scores of from 2.9 to 1.

Headaches resolved in all but one case, headache in that patient improving. Six patients had residual evidence of disease, mainly resulting from cerebral nerve involvement. A 79-year-old patient with HP with ANCA died of pneumonia and renal failure 12 years after onset. A 68-year-old patient with idiopathic HP died of pneumonia while receiving immunosuppressive treatment.

All three patients with idiopathic HP recurred with headaches. Moreover, two of these patients had high WBC counts and CRP concentrations at the time of recurrence. PSL was continued at a dosage of 0.06–0.20 mg/kg/day at the time of relapse. Patients whose PSL dosage had been reduced to 0.06 mg/kg/day to manage adverse effects received other immunosuppressive drugs to prevent relapse. The mean PSL dosage in the three patients who relapsed was 0.13 mg/kg/day, whereas in the four non-relapsing patients it was 0.21 mg/kg/day. The prednisone dosage of patients with recurrences were increased after hospitalization. All patients were discharged home with improvement in symptoms and imaging findings.

## Discussion

In this study, we demonstrated that: (1) both patients with idiopathic and secondary HP initially respond well to PSL, their activities of daily living (ADL) improving; (2) no relapses occurred while receiving moderate dosages of PSL (15–30 mg); and (3) recurrences occurred in patients with idiopathic HP when the PSL dose was reduced to 15 mg or less.

All eight patients responded well to early immunotherapy, except for one with an acute course who died quickly. Our patients’ responses to initial treatment with HP were consistent with those previously reported.^18,19^ Few studies have examined the effects of treatment on ADL objectively. In our study, the mean ADL assessed by mRS improved from 2.9 to 1. In particular, headaches responded well to immunotherapy. Most sequelae were related to cranial nerve involvement. These results indicate that immunotherapy can both stop progression and improve ADL scores in patients with HP. Early treatment on the basis of a precise diagnosis is desirable to minimize sequelae.

No relapses occurred during treatment with moderate doses of PSL (15–30 mg). Conversely, as we have previously reported,^19^ PSL doses of ≤20 mg are closely associated with recurrence in patients with idiopathic HP. These findings suggest that PSL has a dose-dependent effect on prevention of relapse inhibition but does not result in improvements in some patients with HP. The clinical course of HP is monophasic in approximately 30% of patients, progressive in approximately 20%, and relapsing-remitting in approximately 40%. Approximately two-thirds of patients with IgG4-related HP have monophasic clinical courses, indicating that the prognosis of this group is relatively good. In contrast, approximately 40% of patients with idiopathic HP or HP with ANCA present with relapsing-remitting disease, which is a larger proportion than for IgG4-related HP (approximately 20%).^1^ Yokoseki et al. have reported that recurrences tend to occur within 1 year of disease onset.^24^

Idiopathic HP, the cause of which is not known at the time of diagnosis by definition, can be considered to be a group of diseases with a variety of pathologies.^8^ Most patients with idiopathic HP respond to immunotherapy and histological examination shows fibrotic thickening of the dura mater and inflammatory cell infiltration, mainly with lymphocytes. Some type of autoimmune mechanism is therefore strongly suspected; however, the nature of such a mechanism has not yet been established. The mechanism for hypertrophy of the dura mater in idiopathic HP has also not yet been determined. However, measuring high-sensitivity ANCA and performing IgG4 immunostaining of the affected dura mater are expected to elucidate the factors that affect the varying course of idiopathic HP and its responses to PSL. Cui et al. reported that, in patients with IgG4-related HP, the transforming growth factor-β1/SMAD2/SMAD3 pathway is critical and is thus a potential novel therapeutic target.^25^ Interestingly, irbesartan reportedly abolished dural inflammatory cell infiltration and fibrotic thickening in all treated LATY136F mice, this being accompanied by reduced transforming growth factor-β1 and non-phosphorylated and phosphorylated SMAD2/SMAD3.^25^ Further elucidation of the pathogenesis of idiopathic HP is necessary: accurate understanding of its pathogenesis may lead to development of better therapeutic strategies.

In this study, four patients had diffuse-type and four focal-type dural thickening patterns according to MRI. Intriguingly, all three patients with HP with ANCA had focal-type dural thickening, whereas diffuse-type dural thickening occurred more frequently in those with idiopathic HP. In a previous study, one of two patients with HP with ANCA (50%) had focal dural thickening, whereas 9 of 11 with idiopathic HP (81.8%) had diffuse dural thickening.^22^ Differences in the pattern of dural lesions may be useful in determining the pathogenesis of HP but have not been found to be associated with prognosis or response to treatment. In addition, it is unclear whether diffuse versus localized dural thickening is associated with immunological differences. Future studies are awaited.

In our three patients with idiopathic HP, exacerbation of headache was the first indicator of recurrence. Additionally, two of them had increased inflammatory markers. However, intensifying immunotherapy after development of symptoms and abnormalities in blood tests did not always achieve a good therapeutic outcome. Therefore, earlier detection of recurrence is desirable. Because of the adverse effects of contrast-enhanced MRI, it may be preferable to assess dural thickening on low signal T1-weighted images on plain MRI when reducing the PSL dosage to ≤20 mg/day. In forms of HP other than idiopathic HP, systemic symptoms or abnormal laboratory findings rather than the symptoms of HP may suggest disease activity, making it easier to achieve control of these forms of the disease than of idiopathic HP, in which there are few indicators of disease activity. Further studies are needed to develop means of early detection of recurrence in patients with idiopathic HP.

Yokoseki et al. found that HP with ANCA tends to be limited to the central nervous system, similarly to ophthalmic-, pulmonary-, or renal-limited vasculitis. Moreover, PSL plus cyclophosphamide may achieve better outcomes than PSL alone.^24^ Two of our three patients with HP with ANCA cases had the limited central nervous system form. However, one of them died of renal failure after 12 years of treatment, probably as a result of MPO-ANCA-related nephritis. We did not perform a renal biopsy. This patient did not receive immunosuppressive drugs because of age. Additional prospective studies on patients with HP with ANCA are needed to determine whether the lesions are confined to the central nervous system even in the long term and how to optimize treatment in older patients.

Despite several new treatments for HP having recently been developed,^25,26^ the current treatments are not very effective. Further progress is therefore needed.

### Limitations

This study was retrospective and selection of medication and timing of dose reduction depended on the judgment of the principal neurologists. The duration of follow-up and intervals between MRIs and blood tests varied from case to case. In addition, this was a relatively small study because HP is so rare. However, we believe that our findings regarding (1) the relationship between relapse and low PSL dosage, (2) clinical and laboratory findings at the time of relapse, and (3) patients with HP with ANCA showing systemic abnormalities during long-term follow-up will provide useful information for future studies.

## Conclusions

Patients with idiopathic HP and HP associated with immune disorders respond well to steroids and immunosuppressive drugs. However, PSL dosage reduction is associated with a high rate of relapse, mainly in patients with idiopathic HP. Further elucidation of the pathogenesis of idiopathic HP and development of treatments that are capable of preventing recurrence are necessary.

## Figures and Tables

**Table1:** Clinical features of the eight study patients with hypertrophic pachymeningitis

Case	Duration of follow up (month)	Age of onset (years)	Sex	Underlying disease	Dura mater thickening on T1WI	Pattern of dural thickening on MRI	Initial symptoms	Headache during entire course	WBC at first visit (3300–8600/μL)	CRP at first visit (<0.14 mg/dl)	ANA at first visit	CSF cell counts on first visit (<5/μL) (mono/poly)	CSF protein on first visit (10–40 mg/dL)	CSF/serum glucose at firat visit (mg/dL)	Steroid pulse	Maximum PSL daily dose (mg/kg/day)	PSL maintenance daily dose (mg/kg/day)	Immuno-suppressive drug	Drug treatment terminated	mRS before treatment	mRS after treatment	Sequelae	Recurrence	Symptoms at recurrence	WBC at recurrence (3300–8600/μL)	CRP at recurrence (<0.14 mg/dL)	Died
1	52	68	M	Idiopathic	+	Diffuse	Dizziness, diplopia, defaness	+	4900	1.2	Negative	27 (27/0)	58	55/98	+	0.90	0.06	+(CPA)	No	4	1	Dizziness	+	Headache, dizziness, diplopia	5900	<0.3	+(pneumonia)

2	14	42	F	Idiopathic	+	Focal	Headache, left facial pain and sensory disturbance, diplopia, defaness	+	10100	1.5	Homogeneous ×20	10 (6/4)	69	69/92	+	1.07	0.15	–	No	4	1	Dysesthesia	+	Headache, left facial pain	10100	2.9	–

3	53	34	F	Idiopathic	+	Diffuse	Headache, dysarthria, dysphasia	+	10700	4.1	Positive	6 (6/0)	71	70/105	–	0.50	0.20	–	No	2	1	None	+	Headache	11100	3.12	–

4	144	79	F	MPO-ANCA-RV	+	Focal	Headache, right facial pain and palsy, diplopia, defaness	+	10000	14.6	Negative	9 (6/3)	44	62/98	+	0.90	0.36	–	No	2	1	Visual field defect	–	—	—	—	+(pneumonia, renal failure)

5	152	73	M	MPO-ANCA-RV	+	Focal	Headache, dizziness, left ptosis	+	9800	1.9	Negative	3 (3/0)	28	61/110	–	0.74	0.10	–	No	2	1	Headache	–	—	—	—	–

6	21	77	F	MPO-ANCA-RV	+	Focal	Headache, diplopia, left ptosis	+	11400	8.9	Negative	8 (6/2)	59	53/102	+	0.82	0.26	+(AZA)	No	3	1	None	–	—	—	—	–

7	18	29	F	IgG4-RD	+	Diffuse	Headache, diplopia, deafness	+	6200	2.18	Negative	17 (14/3)	48	61/77	+	0.50	0.20	+(AZA)	No	2	1	Deafness	–	—	—	—	–

8	20	64	F	Ulcerative colitis	+	Diffuse	Headache, diplopia, left vision disturbance	+	13400	3.33	Homogeneous ×40	42 (22/20)	75	66/113	+	0.88	0.13	–	No	4	1	Visual field defect	–	—	—	—	–

ANA: antinuclear antibodies, AZA: azathioprine, CPA: cyclophosphamide, CSF: cerebrospinal fluid, F: female, IgG4-RD: immunoglobulin G 4 related diseases, M: male, MPO-ANCA-RV: myeloperoxidase antineutrophil cytoplasmic antibody related vasculitis, mRS: modified Rankin scale, PSL: prednisolone, T1WI: T1 weighted imaging
